# Enhanced Data-Processing Algorithms for Dispersive Interferometry Using a Femtosecond Laser

**DOI:** 10.3390/s24020370

**Published:** 2024-01-08

**Authors:** Tao Liu, Hiraku Matsukuma, Amane Suzuki, Ryo Sato, Wei Gao

**Affiliations:** Precision Nanometrology Laboratory, Department of Finemechanics, Tohoku University, Sendai 980-8579, Japan; liu.tao.q8@dc.tohoku.ac.jp (T.L.); amane.suzuki.t5@dc.tohoku.ac.jp (A.S.); ryo.sato.b8@tohoku.ac.jp (R.S.); i.ko.c2@tohoku.ac.jp (W.G.)

**Keywords:** absolute distance measurement, dispersive interferometry, inverse Fourier transform

## Abstract

Dispersive interferometry based on a femtosecond laser is extensively utilized for achieving absolute distance measurements with high accuracy. However, this method cannot measure arbitrary distances without encountering a dead zone, and deviations in its output results are inevitable due to inherent theory limitations. Therefore, two enhanced data-processing algorithms are proposed to improve the accuracy and reduce the dead zone of dispersive interferometry. The principles of the two proposed algorithms, namely the truncated-spectrum algorithm and the high-order-angle algorithm, are proposed after explaining the limitations of conventional methods. A series of simulations were conducted on these algorithms to show the improved accuracy of measurement results and the elimination of the dead zone. Furthermore, an experimental setup based on a dispersive interferometer was established for the application of these proposed algorithms to the experimental interference spectral signals. The results demonstrated that compared with the conventional algorithm, the proposed truncated-spectrum algorithm could reduce the output distance deviations derived from direct inverse Fourier transforming by eight times to reach as low as 1.3 μm. Moreover, the unmeasurable dead zone close to the zero position of the conventional algorithm, i.e., the minimum working distance of a dispersive interferometer, could be shortened to 22 μm with the implementation of the proposed high-order-angle algorithm.

## 1. Introduction

Length is one of the seven international standard quantities, and its measurement with high resolution is an important issue in the field of dimensional metrology. The employment of Michelson interferometry has made it possible to determine sub-micrometer displacements [1,2,3]. However, this method can only determine the relative distance variations and lacks the capability to directly output the absolute distance [4,5,6]. Since its inception in the late 20th century, the optical frequency comb (OFC), which can provide numerous ultra-narrow linewidth wavelengths over a broad optical spectral range, has been widely used in the areas of optical frequency [7,8], angles [9,10,11,12], spectroscopy [13], etc. Furthermore, versatile absolute distance measurement methods have been developed based on the OFC, encompassing synthetic wavelength interferometry (SWI) [14,15,16], multi-wavelength interferometry (MWI) [17,18,19], dispersive interferometry [20,21,22], dual-comb interferometry [23,24,25], and time-of-flight (TOF) measurement [26,27,28,29]. Among these methods, dispersive interferometry, which utilizes the interferogram across a broad spectrum, is distinguished from other methods by its capability of achieving high-resolution distance measurements. However, it still exhibits certain deficiencies, particularly the presence of a dead zone where distance measurements are not feasible using this method. Considering the mismatch between the limited wavelength resolution of the conventional optical spectrum analyzer (OSA) and the general repetition frequency (sub-GHz) of OFC, it is not possible to analyze each mode of OFC using a conventional OSA. Therefore, there is a maximum measurable distance *l*_max_, which is the Nyquist sampling limit for a dispersive interferometer using an OSA with a resolution of ∆*λ*. The maximum measurable distance can be described as *l*_max_ = *λ*^2^/4*n*∆*λ*, where *λ* and *n* represent the central wavelength and the refractive index, respectively.

Several researchers have dedicated considerable effort to extending the maximum working distance of dispersive interferometry. These endeavors can be broadly categorized into two theoretical groups: one group has focused on enhancing the resolution of the spectrometer, while the other group has concentrated on reducing the interference signal or the comb density in front of the spectrometer. Van den Berg et al. improved the resolution of a spectrometer to 50 GHz, based on a virtually imaged phase array and grating, and finally increased the non-ambiguity of a dispersive ranging system to 15 cm [30]. Joo et al. proposed the use of a Fabry–Perot etalon to decrease the mode density of the interference signal before it enters the spectrometer, resulting in a non-ambiguity range of 1.46 mm. Recently, advancements in extending the maximum working distance have been achieved with the development of OFC sources with a higher repetition frequency [31]. Jang et al. developed a spectrally resolved interferometer powered by a soliton microcomb with a repetition frequency of 88.5 GHz, which could extend the non-ambiguity range over 23 mm with a receptivity of 3 nm [32]. However, in their research, the experimental interference signal was directly subjected to inverse Fourier transform, resulting in a broad pulse in the time domain. Consequently, a polynomial fitting method was required to extract the time delay with high accuracy. Similarly, Wang et al. proposed a chip-scaled soliton microcomb with a repetition frequency of 48.97 GHz, which could increase the performance of traditional optical frequency-based dispersive interferometry and extend the ambiguity-resolved distance up to ~3.063 mm [33]. Nevertheless, the accuracy of time-domain peak detection in this research was also constrained, and a three-point fitting method was indispensable in locating the time peak precisely. It is noteworthy that the other aspect of the dead zone, which is the minimum measurable distance *l*_min_, seemed to be ignored. 

In dispersive interferometry, the time delay *τ* caused by a target distance is embedded within the cosine component of a spectral interference signal, which is *I*(*f*) = cos(2π*fτ*). The employment of inverse Fourier transform is an effective approach to unveiling the time delay from the spectral signal in the frequency domain. According to the principles of Fourier transform theory, at least one integer cosine period is required to obtain accurate inverse Fourier transform results [34,35]. Therefore, for distances shorter than the minimum working distance *l*_min_, the interference signal becomes too sparse to contain even one complete cosine period, resulting in the inability of inverse Fourier transform to obtain accurate results. Hence, research on eliminating the minimum working distance *l*_min_ is a critical issue in micrometer-order short-range absolute distance measurement. Niu et al. made great contributions to analyzing the limitations of dispersive interferometry [36,37]. Based on the Nyquist sampling condition, the resolution of the time delay is determined by the reciprocal of the spectral width, giving rise to the measurement results by dispersive interferometry characterized as a step-like pattern as the target distance increases. Therefore, the occurrence of measurement deviations in dispersive interferometry is inevitable and can only be mitigated by employing a source with a broad-spectrum width, making it pivotal to eliminate the systematic error through improvements in the data-processing procedure of dispersive interferometry. 

In this paper, after clarifying the mechanism for generating the time-peak error and the dead zone in the conventional data-processing algorithm for dispersive interferometry, two enhanced data-processing algorithms are proposed, which can increase the output accuracy and shorten the dead zone to close to the zero position of measurement, i.e., the minimum working distance of dispersive interferometry, respectively. The first algorithm is named the truncated-spectrum algorithm and the second one is known as the high-order-angle algorithm. The feasibility of the proposed algorithms was verified by simulation and experiment through comparison with the results of the conventional algorithm.

## 2. Principles

### 2.1. Principle of the Truncated-Spectrum Algorithm

Dispersive interferometry using a femtosecond laser is typically realized using a Michelson-interferometer-type configuration, in which the optical path difference (OPD) between the reference and measurement arms can be precisely ascertained by analyzing the interference spectrum with an appropriate data-processing algorithm. In this subsection, we give an overview of the conventional data-processing algorithm, based on which, an enhanced data-processing algorithm, referred to as the truncated-spectrum algorithm, is proposed.

**Figure 1:**
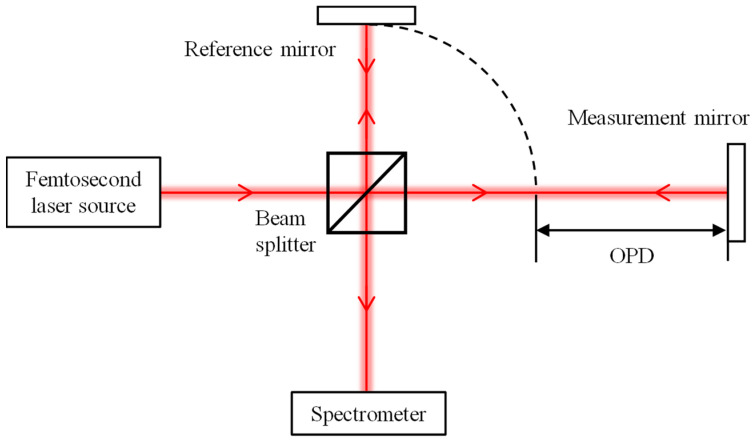
Optical configuration of the dispersive interferometer.

Figure 1 depicts the optical setup of a dispersive interferometer. A laser beam emitting from a femtosecond laser source is first split by a beam splitter into two separate paths. One beam is directed towards a reference mirror, while the other is reflected by a measurement mirror. Subsequently, the two beams are recombined and interfere with each other, resulting in an interference signal. This signal is ultimately detected and analyzed by a spectrometer. For simplicity, the *k*th spectral output of a spectrometer is assumed to correspond to the frequency *f_k_* and the wavelength *λ_k_*. In theory, the intensity of the spectral interference signal output from the spectrometer is expressed as follows:

(1) $$I(f_{k})=S(f_{k})\cdot \left[1+A\cdot \cos\left(2\pi f_{k}\tau \right)\right],$$

where *S*(*f_k_*) is the intensity of the femtosecond laser source, *A* is a parameter representing the intensity difference in the reference and measurement beam, and *τ* is the time delay caused by the optical path difference 2*n*(*f*)*L* between the reference beam and the measurement beam. The time delay *τ* can be explicitly given as *τ* = 2*n*(*f*)*L*/*c*, where *n* and *c* are the refractive index of air and light speed in a vacuum, respectively.

In the conventional data-processing algorithm [31], the spectral interference signal is directly inverse Fourier transformed into a time-domain function *i*(*t*) as follows:

(2) $$i\left(t\right)=s\left(t\right)⊗\left[\delta \left(t\right)+\frac{1}{2}\delta \left(t-\tau \right)+\frac{1}{2}\delta \left(t+\tau \right)\right]=s\left(t\right)+\frac{A}{2}\cdot S\left(t-\tau \right)+\frac{A}{2}\cdot S\left(t+\tau \right),$$

where δ(t) is a unit impulse function and *s*(*t*) is the inverse Fourier transform of source spectrum *S*(*f*). Assuming the femtosecond laser source has a Gaussian-like spectrum, both *S*(*f*) and *s*(*t*) have Gaussian-like shapes. Three Gaussian-like pulses can then be observed in *i*(*t*), with their peaks located at −*τ*, 0, and *τ*, respectively. The pulse of *τ* is then extracted using a time window centered at *τ*. It is worth mentioning that the overlap of two pulses located at 0 and *τ* can remarkably deteriorate the minimum working distance of the dispersive interferometer. To break through this limitation and shorten the dead zone, the authors previously proposed the spectral-fringe algorithm which can remove the central pulse and sharpen the width of Gaussian-shaped time pulses into impulse-shaped ones [38]. After removing the upper and lower envelopes of the detected spectral interference signal in Equation (1), a modified spectral interference signal *I_m_*( $f_{k}$ ) can be obtained by:

(3) $$I_{m}\left(f_{k}\right)=A\cdot \cos\left(2\pi f_{k}\tau \right)=\frac{I\left(f_{k}\right)}{S\left(f_{k}\right)}-1.$$

Hence, the inverse Fourier transforming of spectral interference signal is regenerated as the modified time function *i_m_*(*t*):

(4) $$i_{m}(t)=FT^{-1}\left[I_{m}\left(f_{k}\right)\right]=\frac{A}{2}\cdot \delta \left(t+\tau \right)+\frac{A}{2}\cdot \delta \left(t-\tau \right).$$

By the principles of Fourier transform theory, the resolution in the time domain *t*_res_ is dominated by the reciprocal of frequency width and can be expressed as follows:

(5) $$t_{\mathrm{res}}=\frac{1}{f_{\mathrm{width}}}=\frac{1}{f_{2}-f_{1}},$$

where *f*_width_ is the width of the spectral signal, and *f*_1_ and *f*_2_ are the minimum and maximum spectral frequency, respectively. Based on the equality of *L* = *t*∙*c*/2, it is easy to deduce that the resolution of output distance, denoted as *L*_res_, can be calculated as follows:

(6) $$L_{\mathrm{res}}=\frac{c}{2}\cdot \frac{1}{f_{2}-f_{1}}=\frac{1}{2}\cdot \frac{\lambda_{1}\times \lambda_{2}}{\lambda_{1}-\lambda_{2}},$$

in which *λ*_1_ and *λ*_2_ represent the corresponding wavelength of the minimum and maximum spectral frequency, calculated by *λ* = *c*/*f*. Many researchers have reached this conclusion, and consider the output results from dispersive interferometry to be step-shaped due to the existence of *L*_res._ In other words, the attainable results by dispersive interferometry are limited to integer times of *L*_res_. Therefore, the error between the output results and the real distance is inevitable, and its value varies within the interval of [− $\frac{1}{2}L_{\mathrm{res}}$ , + $\frac{1}{2}L_{\mathrm{res}}$ ] [32,33,36]. The only way to mitigate this error is to increase the spectral width to reach a smaller value for the distance resolution *L*_res_.

However, a different perspective arises when we revisit the principles of discrete Fourier transform, which requires us to utilize integer periods of a cosine function to perform Fourier transform or inverse Fourier transform to ensure its correctness [34]. Therefore, the spectral signal should be truncated with integer periods before performing inverse Fourier transform in dispersive interferometry. Assuming this truncation process is operated at a constructive interference position, the interference signal reaches its local maximum. The width of the truncated spectral signal can be expressed as: ${f^{\prime }}_{\mathrm{width}}={f_{2}}^{\prime }-{f_{1}}^{\prime }$ , in which ${f_{1}}^{\prime }$ and ${f_{2}}^{\prime }$ are the minimum and maximum frequency of the truncated spectral signal, respectively, and should satisfy:

(7) $$L=\frac{1}{2}\cdot n\cdot \frac{c}{{f_{1}}^{\prime }}=\frac{1}{2}\cdot \left(n+k\right)\cdot \frac{c}{{f_{2}}^{\prime }},$$

where *L* represents the target distance, *n* is an arbitrary positive integer number, and *k* is the period number of the truncated spectrum. Meanwhile, integer *n* can be calculated by:

(8) $$n=k\cdot \frac{{\lambda_{2}}^{\prime }}{{\lambda_{1}}^{\prime }-{\lambda_{2}}^{\prime }}.$$

It is important to highlight that, in the case of a truncated spectrum, its frequency width ${f^{\prime }}_{\mathrm{width}}$ , as well as the minimum and maximum frequency ${f_{1}}^{\prime }$ and ${f_{2}}^{\prime }$ , are not constant and vary with the target distance, leading to the distance resolution ${L^{\prime }}_{\mathrm{res}}$ , which is a variable parameter and can be expressed as:

(9) $${L^{\prime }}_{\mathrm{res}}=\frac{c}{2}\cdot \frac{1}{{f_{2}}^{\prime }-{f_{1}}^{\prime }}=\frac{1}{2}\cdot \frac{{\lambda_{1}}^{\prime }\times {\lambda_{2}}^{\prime }}{{\lambda_{1}}^{\prime }-{\lambda_{2}}^{\prime }}.$$

Meanwhile, the value of time resolution ${t^{\prime }}_{\mathrm{res}}$ can be calculated by:

(10) $${t^{\prime }}_{\mathrm{res}}=\frac{1}{{f^{\prime }}_{\mathrm{width}}}=\frac{1}{{f_{2}}^{\prime }-{f_{1}}^{\prime }}.$$

Based on Equations (6)–(10), the target distance can be re-generated as:

(11) $$L=\frac{1}{2}\cdot n\cdot \frac{c}{{f_{1}}^{\prime }}=\frac{1}{2}\cdot k\cdot \frac{{\lambda_{1}}^{\prime }\times {\lambda_{2}}^{\prime }}{{\lambda_{1}}^{\prime }-{\lambda_{2}}^{\prime }}=k\cdot {L^{\prime }}_{\mathrm{res}}=\frac{c}{2}\cdot k\cdot {t^{\prime }}_{\mathrm{res}}.$$

In comparison with results obtained without a preliminary truncation of the spectrum, the target distance in this scenario consistently aligns with the integer times of the distance resolution ${L^{\prime }}_{\mathrm{res}}$ and the output distance exhibits a linear increase with the target distance, instead of the step-like pattern observed previously. Moreover, the error of output distance is only determined by the accuracy of the parameter *k*, and distance resolution ${L^{\prime }}_{\mathrm{res}}$ is related to the accuracy of the truncated frequencies ${f_{1}}^{\prime }$ and ${f_{2}}^{\prime }$ , rather than the width of the spectrum signal. 

Furthermore, there is another manifest improvement in the inverse Fourier transform results for the truncated spectrum in comparison with the non-truncated one. In the case of a truncated spectrum, the time pulse in the time domain takes on a sharp impulse shape because the time delay *τ* caused by distance *L* must be an integer multiple of the time resolution ${t^{\prime }}_{\mathrm{res}}$ . Oppositely, the time pulse from a non-truncated spectrum has a broader pulse-like shape due to the fixed time resolution *t*_res_, leading to the real time peak always falling within the interval of [*n*∙*t*_res_, (*n* + 1)∙*t*_res_]. Based on the above theoretical analysis, a truncation process that ensures only integer periods of the interference signal are imparted to the subsequent inverse Fourier transform is beneficial to increasing the accuracy of time delay *τ* with a finite spectrum width.

Once the time pulse in the time domain is obtained by inverse Fourier transforming, it can be easily selected by a time window centered at *τ* and the target distance can be calculated by:

(12) $$L=\frac{1}{2}\cdot c\cdot \tau .$$

Meanwhile, it is also feasible to extract the wrapped phase *φ*(*f*) by operating a Fourier transform to the selected time pulse and finally output the target distance *L* by the first-order derivation of the unwrapped phase *dΦ*(*f*)/*df*, which can be written as follows:

(13) $$I^{\prime }\left(f\right)=\frac{A}{2}\cdot S\left(f\right)\cdot e^{-j2\pi f\tau },$$

(14) φf=tan−1−ImI′fReI′f,

(15) $$L=\frac{c}{4\pi n_{g}}\cdot \frac{d\Phi \left(f\right)}{df}.$$

where $I^{\prime }\left(f\right)$ is the Fourier transform results of the selected time pulse, *φ*(*f*) is the wrapped phase which changes within the range of [−*π*/2, +*π*/2], and *Φ*(*f*) is the unwrapped phase. It is worth noting that, theoretically, there is no difference between the output results of Equations (12) and (15). For simplicity, results calculated by Equation (12) are used in the following paper.

### 2.2. Principle of the High-Order-Angle Algorithm

There is a correlation between the density of interference fringes and the target distance, meaning that the integer periods of the interference signal increase as the target distance increases. For the minimum working distance *l*_min_, there is only one complete integer period in its interference signal. While, if the target distance continuously decreases to a value smaller than *l*_min_, no complete integer period can be found in the interference signal, rendering the subsequent inverse Fourier transform infeasible. Hence, the minimum working distance, i.e., the dead zone of dispersive interferometry is constrained by the spectral width as *l*_min_ = *c*/(2∙*f*_width_). Coincidentally, this value is equal to the distance resolution outlined in Equation (6). In an actual experiment, it is impossible to measure the infinite spectral width by dispersive interferometry, making it impossible to make the dead zone vanish using the conventional algorithm. To reduce the dead zone of dispersive interferometry and extend its minimum working distance to the zero position, a novel data-processing algorithm based on the high-order-angle equation is proposed.

For simplicity, the analysis commences with the lowest-order-angle equation, i.e., the double-angle equation. This equation illustrates the relationship between the cosine function of the double angle 2*θ* and the original angle *θ* as: cos2*θ* = 2(cos*θ*)^2^ − 1, which can be deduced from the trigonometric function transformation. Notably, the period of cos*θ* is twice as long as that of cos2*θ*. This characteristic can also be extended to the modified spectral interference signal *I_m_*( $f_{k}$ ), whose period can also be doubled by:

(16) $$I_{m}{\left(f_{k}\right)}_{\mathrm{double}}=\cos\left(2\cdot 2\pi f_{k}\tau \right)=2\cdot \cos^{2}\left(2\pi f_{k}\tau \right)-1,$$

in which the period of *I_m_*( $f_{k}$ )_double_ is halved compared to the original modified spectral interference signal *I_m_*( $f_{k}$ ), making the integer period number also double within a certain frequency range. In this situation, the interference signal for the original minimum working distance *l*_min_ exhibits two complete integer periods. As the target distance further decreases to *l*_min_/2, the integer period value of the interference signal decreases to one, which is the minimum threshold for performing inverse Fourier transform. Consequently, the minimum working distance, after being subjected to the folding effect of the double-angle equation, is reduced by half compared to its original value. In other words, *l*_min-double_ = *l*_min_/2.

Moreover, the triple-angle equation of cosine function can also be employed to further reduce the dead zone continuously. The period of the modified spectral interference signal *I_m_*( $f_{k}$ ) can undergo a tripling process as follows:

(17) $$I_{m}{\left(f_{k}\right)}_{\mathrm{triple}}=\cos\left(3\cdot 2\pi f_{k}\tau \right)=4\cdot \cos^{3}\left(2\pi f_{k}\tau \right)-3\cdot \cos\left(2\pi f_{k}\tau \right).$$

Compared with the original modified spectral interference signal *I_m_*( $f_{k}$ ), integer periods of the interference signal in *I_m_*( $f_{k}$ )_triple_ are multiplied by a factor of three. Hence, the minimum working distance experiences a threefold reduction, expressed as *l*_min-triple_ = *l*_min_/3. Based on the theoretical analysis of trigonometric function transformations, the relationship between the cosine function of *n* times angle *n∙θ* and the original angle *θ* can be established as follows:

(18) $$\cos\left(n\cdot \theta \right)=\sum_{k=0}^{\frac{n}{2}}{\left(-1\right)}^{k}\left(\begin{array}{c} n\\ 2k \end{array}\right)\sin^{2k}\theta \cos^{n-2k}\theta .$$

Therefore, by increasing the integer period of the interference signal by a factor of *n*, the corresponding minimum working distance for dispersive interferometry can be reduced by *n* times to *l*_min-n_ = *l*_min_/*n*. The minimum working distance can be compressed to nearly zero using a sufficiently large parameter *n*. In theory, the dead zone of dispersive interferometry, especially for the minimum working distance, can be eliminated through the implementation of this proposed data-processing algorithm, named the high-order-angle algorithm.

## 3. Simulation and Experiment Results

### 3.1. Simulation Results

**Figure 2:**
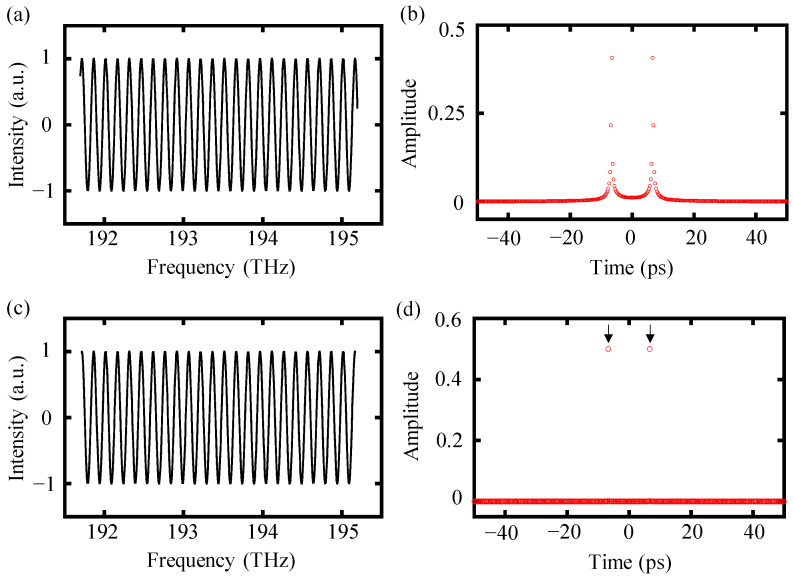
A comparison of inverse Fourier transform results of the truncated-spectrum algorithm and the conventional algorithm. (**a**) The original interference signal *I_m_*( $f_{k}$ ), (**b**) inverse Fourier transform results of *I_m_*( $f_{k}$ ), (**c**) the original interference signal *I_m_*( $f_{k}$ ) is truncated by integer cosine periods, and (**d**) inverse Fourier transform results of the truncated spectrum.

A modified interference spectrum *I_m_*( $f_{k}$ ) defined in Equation (3) with a target distance of 1 mm and a spectrum ranging from 191.7 THz to 195.2 THz was exploited in a simulated data-processing procedure to present the advantages of the proposed truncated-spectrum algorithm in increasing the accuracy of dispersive interferometry. The truncation process was performed by determining the peak positions of the interference signal using “findpeaks” function in MATLAB, which can output the values and positions of local maxima in a set of data. Consequently, the peak positions of the interference signal were ascertained based on the identified positions of local maxima. Figure 2 illustrates a comparative analysis of the inverse Fourier transform results between the truncated spectrum and the non-truncated one.

For the original interference signal *I_m_*( $f_{k}$ ), a direct inverse Fourier transform yielded a broad pulse in the time domain, as shown in Figure 2b. This broad pulse veiled the time delay caused by the target distance of 1 mm, and the position of this time delay was located in the vicinity of the peak in the time domain. In other words, the time delay did not coincide with the peak in the time domain, making it unfeasible to determine the value of the time delay by the position of the time peak directly. Therefore, the utilization of complementary techniques like curve fitting was essential to determine the time delay with high accuracy. In contrast, for the inverse Fourier transform of a truncated spectrum, the time pulse was sharper and narrower with an impulse-like shape and the time delay overlapped with the location of the peak in the time domain, as shown in Figure 2d. Therefore, the impulse-shaped pulse at *τ* could be selected more easily and precisely by a time window for calculating the target distance based on Equation (12), eliminating the requirement for other complementary steps. 

**Figure 3:**
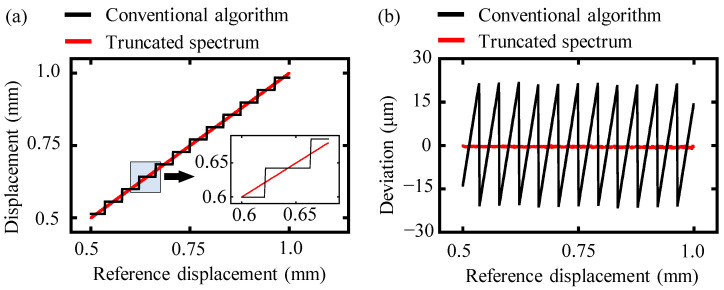
A comparison of simulation results with the truncated-spectrum and the conventional algorithm. The black and magenta lines represent the results of the truncated-spectrum and the conventional algorithm, respectively. (**a**) Simulation displacement and (**b**) deviation of the simulation displacement from the reference displacement.

To further illustrate the benefits of the proposed truncated-spectrum algorithm, another simulation was conducted covering a range of distances from 0.5 mm to 1 mm. The spectrum spanned from 191.7 THz to 195.2 THz, resulting in a spectral width of 3.5 THz and a distance resolution of 43 μm. A comparison of simulation results using the truncated-spectrum algorithm and the conventional algorithm is shown in Figure 3.

As shown in Figure 3a, the simulation displacement showed a step shape for the conventional algorithm with an increasing reference displacement, while the results of the truncated-spectrum algorithm showed a good agreement for the reference displacement. The simulation deviation from the reference displacement is compared in Figure 3b, in which the deviations of conventional algorithm fluctuated within the interval of [−21.5 μm, +21.5 μm] for the theoretical analysis, while the absolute average deviation of the truncated-spectrum algorithm was only 0.5 μm, more than 20 times smaller than that of the conventional one, verifying that the proposed truncated-spectrum is an effective and necessary approach to increasing the accuracy of dispersive interferometry. It should be noted that the simulation deviation of the truncated-spectrum algorithm was mainly caused by the deviations of the starting and ending frequency during the truncation process, which can be alleviated by increasing the sampling frequency.

**Figure 4:**
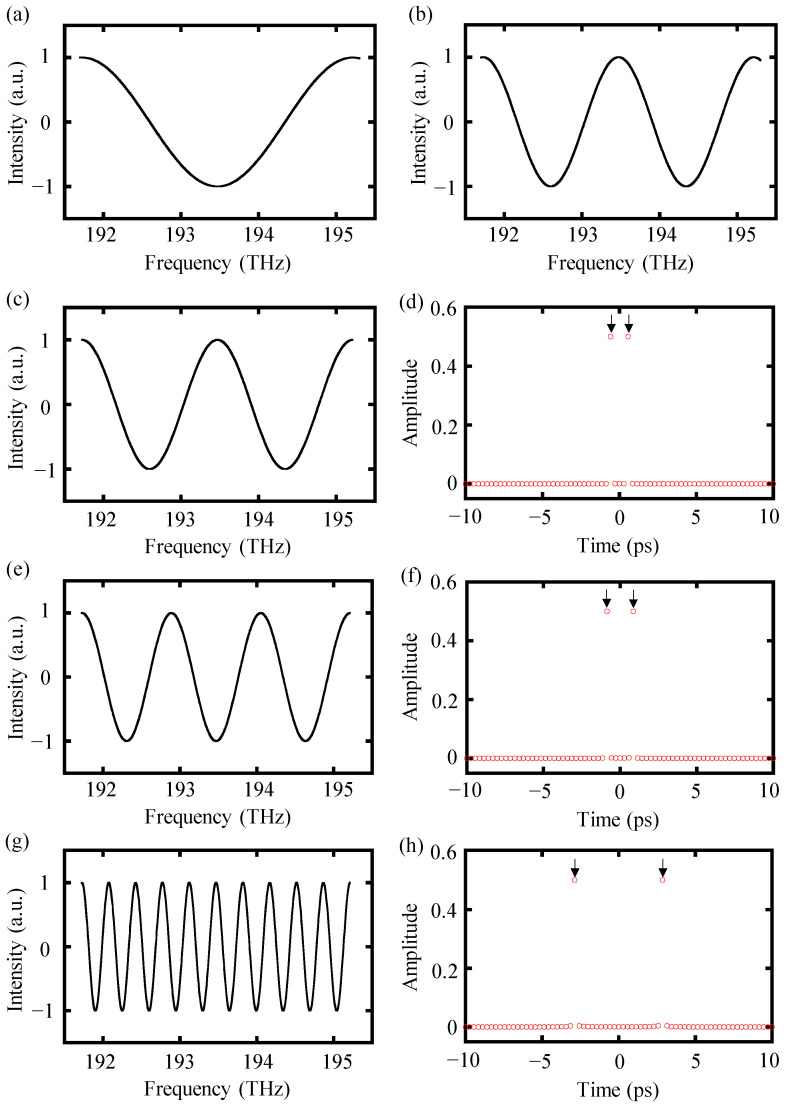
The data-processing procedure of the high-order-angle algorithm. (**a**) Original modified spectral interference signal *I_m_*( $f_{k}$ ) with a distance of 43 μm, with the spectral frequency starting from 191.7 THz and ending at 195.2 THz, (**b**) double-folded interference signal *I_m_*( $f_{k}$ )_double_ using the double-angle equation, (**c**) truncating spectrum into two integer cosine periods, (**d**) inverse Fourier transform result of the truncated spectrum in (**c**), (**e**) truncated triple-folded spectrum, (**f**) inverse Fourier transform of the truncated spectrum in (**e**), (**g**) truncated tenth-folded spectrum, and (**h**) inverse Fourier transform of the truncated spectrum in (**g**).

A modified interference spectrum with a target distance of 43 μm and a spectral width of 3.5 THz was employed in a simulation to present the data-processing procedure of the high-order-angle algorithm. It is worth mentioning that the minimum working distance for dispersive interferometry with a spectral width of 3.5 THz was equal to 43 μm. As with the theoretical analysis, there was only one integer period cosine function in the modified spectral interference signal *I_m_*( $f_{k}$ ). In this simulation, a double-angle algorithm was used to compress the period of *I_m_*( $f_{k}$ ) to the double-folded interference signal *I_m_*( $f_{k}$ )_double_. Subsequently, the folded interference signal was truncated into integer periods to increase the accuracy of the final result. Finally, inverse Fourier transform was performed to achieve two sharp impulse peaks in the time domain, as illustrated in Figure 4a–d. Similarly, the original modified interference spectrum could also be triple- and ten-folded using the triple-angle equation and tenth-order-angle equation, in which more complete integer periods could be generated and the time delay could be easily picked up in the inverse Fourier transform result, as depicted in Figure 4e–h. Finally, the distance could be calculated by Equation (12).

**Figure 5:**
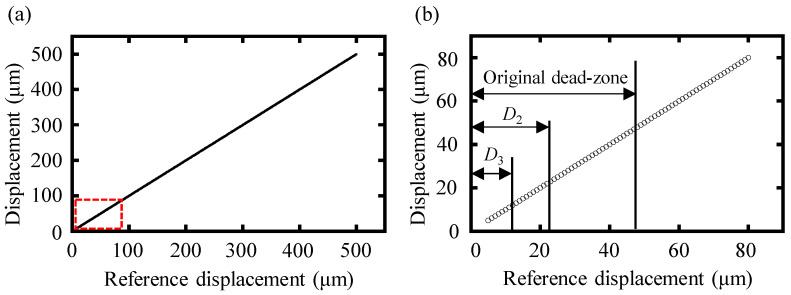
Simulation results of the high-order-angle algorithm. (**a**) Simulation displacement with an increasing reference displacement from 5 μm to 500 μm and (**b**) amplification of the red box highlighted region in the left figure (**a**). *D*_2_ represents the dead zone of the double-angle algorithm and *D*_3_ represents the dead zone of the triple-angle algorithm.

A simulation with an increasing reference displacement from 5 μm to 500 μm was performed to illustrate the effectiveness of the proposed high-order-angle algorithm in eliminating the dead zone, especially the minimum working distance of dispersive interferometry. Simulation results of the high-order-angle algorithm were highly consistent with the reference displacement, as shown in Figure 5a. Furthermore, the small displacement region highlighted in the red box of Figure 5a is magnified in Figure 5b, in which it can be seen that by using the double-angle, triple-angle, and tenth-order-angle algorithm, the dead zone of the dispersive interferometry could be reduced to two times, three times, and ten times that of the original dead zone. Furthermore, the dead zone could be continuously decreased to the near zero region and be eliminated by employing a higher-order-angle algorithm.

### 3.2. Experimental Setup

**Figure 6:**
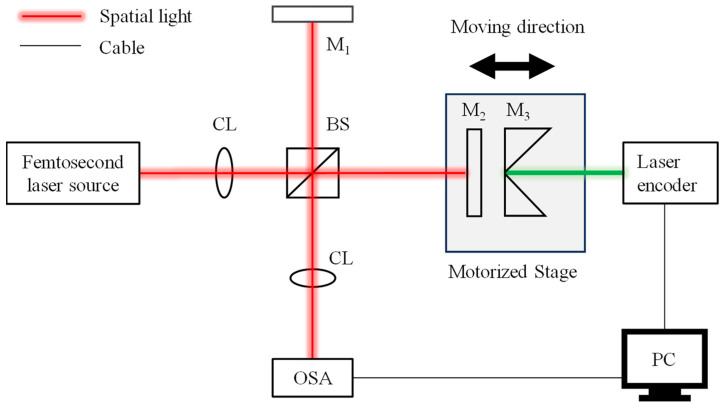
Schematic of the experimental setup, which was a dispersive interferometry using a femtosecond laser. CL: collimating lens, BS: beam splitter, M_1_: reference mirror, M_2_: measurement mirror, M_3_: retroreflector, and OSA: optical spectrum analyzer.

Figure 6 illustrates the optical configuration of the proposed method. A mode-locked femtosecond fiber laser source with a center frequency *f_c_* of 192.175 THz and a repetition frequency *f*_rep_ of 100 MHz was employed for the experiment. The central wavelength of the laser source was 1560 nm and its spectral FWHM was ~62.4 nm. A laser with an approximate power of 5 mW was incident into a beam splitter (OptoSigma NPCH-10-15500, Santa Ana, CA, USA) via a single-mode fiber. The light reflected and transmitted by the beam splitter served as the reference and measurement beams, respectively. These beams were then reflected by two square-protected silver mirrors (Thorlabs PFSQ05-03-P01, Newton, MA, USA). The mirror in the reference beam remained stationary, while the measurement mirror was mounted on a single-axis motorized stage (Saruka Seiki KXC04015-CA, Osaka, Japan) that allowed for linear translation along the optical path within a travel range of ±7 mm. Moreover, the retroreflector for a commercial laser encoder (Renishaw RLD10-3R, Wotton-under-Edge, UK) was also mounted on the motorized stage to monitor the movement of the measurement mirror. The resolution of the laser encoder could achieve tens of picometers, meaning that the displacement of the measurement mirror could be calibrated with high accuracy. The optical interference signal was analyzed by an optical spectrum analyzer (Yokogawa AQ6370D, Tokyo, Japan) with a wavelength resolution of 0.02 nm. 

### 3.3. Experimental Results and Discussion

**Figure 7:**
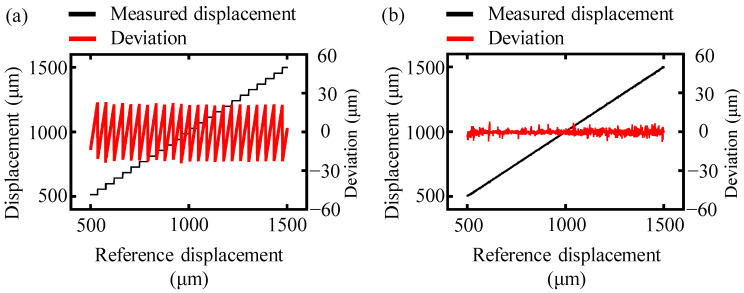
A comparison of the measurement displacement of the conventional and truncated-spectrum algorithms. The black and magenta lines represent the experimentally measured displacement and the deviation from the reference displacement, respectively. (**a**) The measurement displacement and the deviation using the conventional algorithm and (**b**) the measurement displacement and the deviation using truncated-spectrum algorithm.

In the experiment, the target mirror in the measurement arm was moved continuously from 0 to 1.5 mm with a step of 0.1 μm, and the interference signal within the frequency range of 191.7 THz to 195.2 THz was recorded using the OSA at the same time. The laser encoder was set to zero at the initial position to calibrate the movement distance of the motorized stage and the measurement mirror. The detected interference spectrum was processed both by the conventional and the proposed truncated-spectrum algorithm, and the comparison of these measurement results is presented in Figure 7.

The determined displacement of the conventional algorithm showed step-shaped structures as the reference displacement increased, and its corresponding measurement deviation compared to the reference displacement fluctuated within the interval of [−21.5 μm, +21.5 μm] with an average value of 10.7 μm, as shown in Figure 7a. This tendency was similar to the theoretical and simulation analysis. On the other hand, the results obtained by the proposed truncated-spectrum algorithm showed a good agreement with the reference displacement, as depicted in Figure 7b. The average deviation for the truncated-spectrum algorithm was approximately 1.3 μm, which was almost ten times smaller than that of the conventional algorithm. Thus, the proposed truncated-spectrum algorithm was effective in increasing the accuracy of dispersive interferometry.

**Figure 8:**
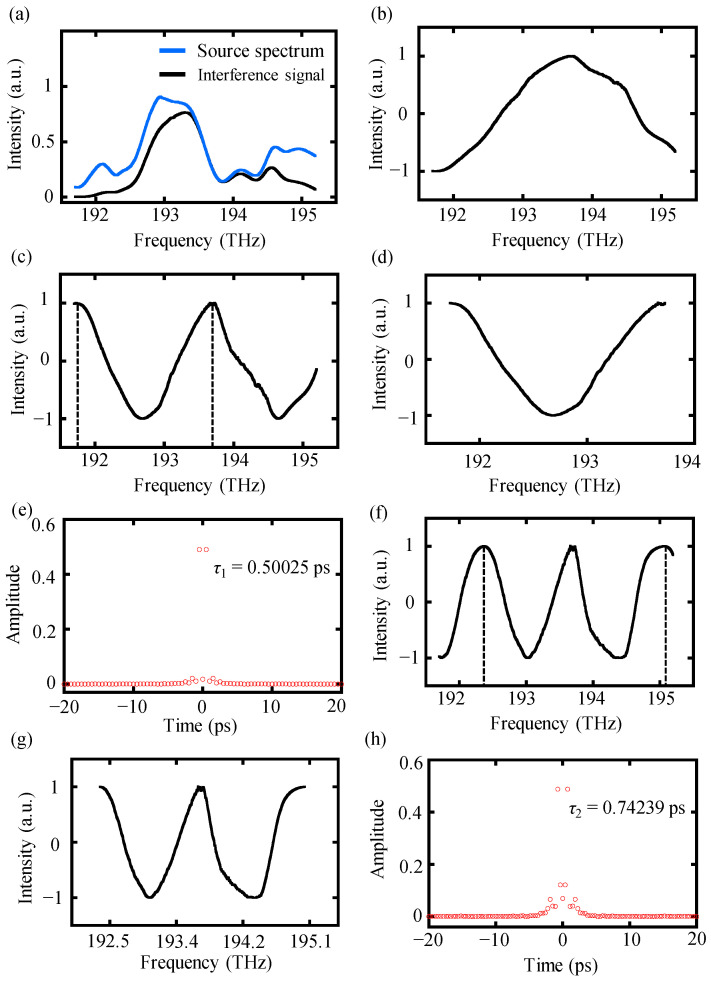
The experimental data-processing procedure of the high-order-angle algorithm. (**a**) The blue line represents the source spectrum and the black line represents the experimental interference spectrum with a target distance of 38.2 μm, (**b**) the modified spectral interference signal *I_m_*( $f_{k}$ ) after preprocessing, (**c**) double-folded interference signal, (**d**) truncating into one integer period and the truncation positions are clarified as the black dash line in (**c**), (**e**) inverse Fourier transform results of the truncated signal, (**f**) triple-folded interference signal, (**g**) the truncated triple-folded interference signal and the truncation positions are clarified as the black dash line in (**f**), and (**h**) inverse Fourier transform results of the truncated signal in (**g**).

Next, another proposed algorithm, that is, the high-order-angle algorithm, is explained. Preprocessing the raw interference signal before performing this high-order-angle algorithm is necessary. An experimental interference spectrum *I*(*f_k_*) with a target distance of 38.2 μm and the laser source spectrum *S*(*f_k_*) is depicted in Figure 8a. After dividing the interference spectrum *I*(*f_k_*) by the source spectrum *S*(*f_k_*), a normalization was utilized to generate the modified spectral interference signal *I_m_*( $f_{k}$ ), which is shown in Figure 8b. Since the target distance of 38.2 μm was smaller than the minimum working distance of 43 μm with a frequency width of 3.5 THz, no complete cosine period could be found in the interference signal, resulting in a failure operation of the conventional algorithm. In the proposed high-order-angle algorithm, the interference signal could be double-folded using the double-angle equation. Subsequently, a truncation procedure was performed to extract integer periods of the spectrum. The truncation points were determined by searching for the peak positions of the spectrum using the “findpeaks” function in MATLAB. Finally, the time delay *τ*_1_ was easily picked up by the inverse Fourier transform of the truncated double-folded signal, as illustrated in Figure 8c–e. Moreover, the experimental interference signal could also be triple-folded using the triple-angle equation, and operating an inverse Fourier transform was required to extract the time delay *τ*_2_, as shown in Figure 8f–h. The measurement results using high-order-angle algorithms, i.e., the double-angle and triple-angle algorithm, were 37.5 μm and 37.1 μm, with deviations of 0.7 μm and 1.1 μm, respectively.

**Figure 9:**
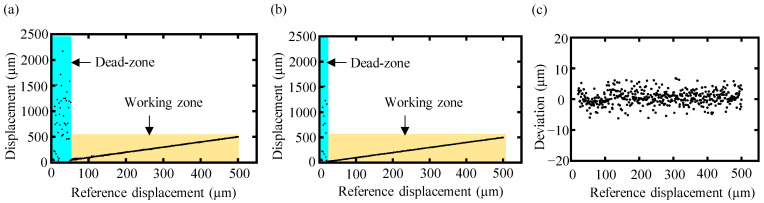
The experimentally measured displacement of the conventional data-processing algorithm and the proposed high-order-angle algorithm with a reference displacement from 0 to 500 μm. (**a**) The measurement displacement of the conventional algorithm, in which the blue region represents the dead zone, and the yellow region represents the working zone. The dead zone of the conventional algorithm was approximately 0–70 μm. (**b**) The measurement displacement of the proposed high-order-angle algorithm. (**c**) The deviation from the reference displacement of the high-order-angle algorithm in its working zone.

A comparison of the measurement results with a reference displacement ranging from 0 to 500 μm obtained both by the conventional data-processing algorithm and the proposed high-order-angle algorithm is presented in Figure 9. With the conventional algorithm, no reasonable results could be outputted for a reference displacement of less than 70 μm, as indicated by the blue region of Figure 9a. On the other hand, reliable measurements by the proposed high-order-angle algorithm were still achievable for target displacements of less than 70 μm, as shown in Figure 9b. By employing the proposed high-order-angle algorithm, the minimum achieved working distance was approximately 22 μm, which indicated that the proposed high-order-angle algorithm could significantly shorten the dead zone of dispersive interferometry to be more than one-third smaller than that of the conventional algorithm, and one-half smaller than the theoretical limitation of the conventional one. The deviation from the reference displacement of the high-order-angle algorithm in its working zone is illustrated in Figure 9c, with an average value of 1.8 μm.

At present, the proposed high-order-angle algorithm is not capable of determining a target distance smaller than 22 μm with high accuracy. This is because, for the interference signal with an ultra-small distance, the corresponding cosine period may only be a quarter or a fifth of a complete one, making it difficult to precisely extract the modified spectral interference signal *I_m_*( $f_{k}$ ) from the original interference signal. It is worth mentioning that an accurately extracted spectral interference signal *I_m_*(*f_k_*) is the cornerstone for operating the proposed high-order-angle algorithm. The employment of the high-order-angle algorithm beyond the triple angle to four or five angles is capable of further shrinking the dead zone to smaller than 22 μm, but only if a distortion-free cosine spectral interference signal *I_m_*( $f_{k}$ ) can be achieved. Otherwise, the utilization of a high-order-angle equation in the proposed algorithm can amplify the error in *I_m_*( $f_{k}$ ) by several times, resulting in significant deviations during the truncation process and affecting the accuracy of the distance measurement outcomes. 

## 4. Conclusions

In this paper, we have introduced two enhanced data-processing algorithms to improve the performance of dispersive interferometry using a femtosecond laser. The first proposed truncated-spectrum algorithm was shown to significantly increase the accuracy of the output results, while the second proposed high-order-angle algorithm effectively reduced the dead zone, particularly the minimum working distance. The principles of the two proposed algorithms were derived, and the simulation work was conducted to illustrate the potential of these algorithms in increasing the accuracy and eliminating the dead zone. An experimental ranging system was established to conduct short-range absolute distance measurements using dispersive interferometry. Measurement results achieved from the conventional data-processing algorithm and the proposed algorithms were compared. It was confirmed that employing the proposed truncated-spectrum algorithm could improve the accuracy of the measurement results by more than eight times compared to the conventional algorithm, resulting in an average deviation of 1.3 μm. Furthermore, the second proposed high-order-angle algorithm was demonstrated to be capable of surpassing the theoretical limitations of the conventional algorithm regarding the dead zone, shortening the dead zone to a minimum of 22 μm, which was at least one-third smaller was achieved by the conventional algorithm.

## Data Availability

The data presented in this study are available on request from the corresponding authors.

## References

[1] Michelson A.A., Pease F., Pearson F. (1929). Repetition of the Michelson-Morley experiment. JOSA.

[2] Gao W. (2010). Precision Nanometrology: Sensors and Measuring Systems for Nanomanufacturing.

[3] Straube G., Fischer Calderón J.S., Ortlepp I., Füßl R., Manske E. (2021). A Heterodyne Interferometer with Separated Beam Paths for High-Precision Displacement and Angular Measurements. Nanomanuf. Metrol..

[4] Gao W., Haitjema H., Fang F.Z., Leach R.K., Cheung C.F., Savio E., Linares J.M. (2019). On-machine and in-process surface metrology for precision manufacturing. CIRP Ann..

[5] Gao W., Kim S.W., Bosse H., Haitjema H., Chen Y.L., Lu X.D., Knapp W., Weckenmann A., Estler W.T., Kunzmann H. (2015). Measurement technologies for precision positioning. CIRP Ann..

[6] Gao W., Ibaraki S., Donmez M.A., Kono D., Mayer J.R.R., Chen Y.-L., Szipka K., Archenti A., Linares J.-M., Suzuki N. (2023). Machine tool calibration: Measurement, modeling, and compensation of machine tool errors. Int. J. Mach. Tools Manuf..

[7] Coddington I., Swann W.C., Newbury N.R. (2008). Coherent multiheterodyne spectroscopy using stabilized optical frequency combs. Phys. Rev. Lett..

[8] Jones D.J., Diddams S.A., Ranka J.K., Stentz A., Windeler R.S., Hall J.L., Cundiff S.T. (2000). Carrier-envelope phase control of femtosecond mode-locked lasers and direct optical frequency synthesis. Science.

[9] Chen Y.L., Shimizu Y., Tamada J., Nakamura K., Matsukuma H., Chen X., Gao W. (2018). Laser autocollimation based on an optical frequency comb for absolute angular position measurement. Precis. Eng..

[10] Shimizu Y., Kudo Y., Chen Y.-L., Ito S., Gao W. (2017). An optical lever by using a mode-locked laser for angle measurement. Precis. Eng..

[11] Chen Y.L., Shimizu Y., Tamada J., Kudo Y., Madokoro S., Nakamura K., Gao W. (2017). Optical frequency domain angle measurement in a femtosecond laser autocollimator. Opt. Express.

[12] Chen Y.L., Shimizu Y., Kudo Y., Ito S., Gao W. (2016). Mode-locked laser autocollimator with an expanded measurement range. Opt. Express.

[13] Fortier T., Baumann E. (2019). 20 years of developments in optical frequency comb technology and applications. Commun. Phys..

[14] Wu G., Takahashi M., Inaba H., Minoshima K. (2013). Pulse-to-pulse alignment technique based on synthetic-wavelength interferometry of optical frequency combs for distance measurement. Opt. Lett..

[15] Doloca N.R., Meiners-Hagen K., Wedde M., Pollinger F., Abou-Zeid A. (2010). Absolute distance measurement system using a femtosecond laser as a modulator. Meas. Sci. Technol..

[16] Minoshima K., Matsumoto H. (2000). High-accuracy measurement of 240-m distance in an optical tunnel by use of a compact femtosecond laser. Appl. Opt..

[17] Wang G., Jang Y.S., Hyun S., Chun B.J., Kang H.J., Yan S., Kim S.W., Kim Y.J. (2015). Absolute positioning by multi-wavelength interferometry referenced to the frequency comb of a femtosecond laser. Opt. Express.

[18] Hyun S., Kim Y.J., Kim Y., Kim S.W. (2010). Absolute distance measurement using the frequency comb of a femtosecond laser. CIRP Ann..

[19] Jin J., Kim Y.J., Kim Y., Kim S.W., Kang C.S. (2006). Absolute length calibration of gauge blocks using optical comb of a femtosecond pulse laser. Opt. Express.

[20] Liang X., Wu T., Lin J., Yang L., Zhu J. (2023). Optical Frequency Comb Frequency-division Multiplexing Dispersive Interference Multichannel Distance Measurement. Nanomanuf. Metrol..

[21] Wu H., Zhang F., Meng F., Liu T., Li J., Pan L., Qu X. (2016). Absolute distance measurement in a combined-dispersive interferometer using a femtosecond pulse laser. Meas. Sci. Technol..

[22] van den Berg S.A., van Eldik S., Bhattacharya N. (2015). Mode-resolved frequency comb interferometry for high-accuracy long distance measurement. Sci. Rep..

[23] Zhu Z., Wu G. (2018). Dual-Comb Ranging. Engineering.

[24] Lee J., Han S., Lee K., Bae E., Kim S., Lee S., Kim S.-W., Kim Y.-J. (2013). Absolute distance measurement by dual-comb interferometry with adjustable synthetic wavelength. Meas. Sci. Technol..

[25] Coddington I., Swann W.C., Nenadovic L., Newbury N.R. (2009). Rapid and precise absolute distance measurements at long range. Nat. Photonics.

[26] Kim W., Jang J., Han S., Kim S., Oh J.S., Kim B.S., Kim Y.J., Kim S.W. (2020). Absolute laser ranging by time-of-flight measurement of ultrashort light pulses. JOSA.

[27] Balling P., Kren P., Masika P., van den Berg S.A. (2009). Femtosecond frequency comb based distance measurement in air. Opt. Express.

[28] Ye J. (2004). Absolute measurement of a long, arbitrary distance to less than an optical fringe. Opt. Lett..

[29] Jang Y.S., Kim S.W. (2018). Distance Measurements Using Mode-Locked Lasers: A Review. Nanomanuf. Metrol..

[30] van den Berg S.A., Persijn S.T., Kok G.J., Zeitouny M.G., Bhattacharya N. (2012). Many-wavelength interferometry with thousands of lasers for absolute distance measurement. Phys. Rev. Lett..

[31] Joo K.N., Kim S.W. (2006). Absolute distance measurement by dispersive interferometry using a femtosecond pulse laser. Opt. Express.

[32] Jang Y.S., Liu H., Yang J., Yu M., Kwong D.L., Wong C.W. (2021). Nanometric Precision Distance Metrology via Hybrid Spectrally Resolved and Homodyne Interferometry in a Single Soliton Frequency Microcomb. Phys. Rev. Lett..

[33] Wang J., Lu Z., Wang W., Zhang F., Chen J., Wang Y., Zheng J., Chu S.T., Zhao W., Little B.E. (2020). Long-distance ranging with high precision using a soliton microcomb. Photonics Res..

[34] Brigham E.O. (1988). The Fast Fourier Transform and Its Applications.

[35] Wang F., Shi Y., Zhang S., Yu X., Li W. (2022). Automatic Measurement of Silicon Lattice Spacings in High-Resolution Transmission Electron Microscopy Images Through 2D Discrete Fourier Transform and Inverse Discrete Fourier Transform. Nanomanuf. Metrol..

[36] Niu Q., Song M., Zheng J., Jia L., Liu J., Ni L., Nian J., Cheng X., Zhang F., Qu X. (2022). Improvement of Distance Measurement Based on Dispersive Interferometry Using Femtosecond Optical Frequency Comb. Sensors.

[37] Niu Q., Zheng J.H., Cheng X.R., Liu J.C., Jia L.H., Ni L.M., Nian J., Zhang F.M., Qu X.H. (2022). Arbitrary distance measurement without dead zone by chirped pulse spectrally interferometry using a femtosecond optical frequency comb. Opt. Express.

[38] Liu T., Wu J., Suzuki A., Sato R., Matsukuma H., Gao W. (2023). Improved Algorithms of Data Processing for Dispersive Interferometry Using a Femtosecond Laser. Sensors.

